# Diagnostic and Therapeutic Properties of Exosomes in Cardiac Fibrosis

**DOI:** 10.3389/fcell.2022.931082

**Published:** 2022-07-04

**Authors:** Jiwen Fan, Meng Ren, Yuquan He

**Affiliations:** ^1^ Department of Cardiology, China-Japan Union Hospital of Jilin University, Changchun, China; ^2^ Department of Medical Oncology, Jilin Provincial Cancer Hospital, Changchun, China

**Keywords:** exosome, cardiac fibrosis, cardiovascular diseases, myofibroblast, miRNAs

## Abstract

Cardiac fibrosis results from both the differentiation of cardiac fibroblasts and excessive accumulation of extracellular matrix (ECM), leading to myocardial stiffness and reduced compliance of the ventricular wall. The conversion of cardiac fibroblasts to myofibroblasts is the most important initiating step in the process of this pathological cardiac remodeling. It occurs during the progression of many cardiovascular diseases, adversely influencing both the clinical course and outcome of the disease. The pathogenesis is complex and there is no effective treatment. Exosomes are extracellular vesicles that mediate intercellular communication through delivering specific cargoes of functional nucleic acids and proteins derived from particular cell types. Recent studies have found that exosomes play an important role in the diagnosis and treatment of cardiac fibrosis, and is a potential biotherapeutics and drug delivery vectors for the treatment of cardiac fibrosis. The present review aimed to summarize the current knowledge of exosome-related mechanisms underlying cardiac fibrosis and to suggest potential therapy that could be used to treat the condition.

## Introduction

Cardiac fibrosis is a pathological process occurring in almost all cardiovascular disorders and results from fibroblast activation and the deposition of excess extracellular matrix (ECM) (Kong et al., 2014; Ma et al., 2018). Cardiac fibrosis leads to increased stiffness and reduced flexibility of the myocardium with varying degrees of myocardial systolic or diastolic dysfunction, resulting in heart failure and death (Li et al., 2018; Maruyama & Imanaka-Yoshida, 2022). Although numerous pathogenic factors have been identified, the specific mechanisms underlying its development are unclear and require further investigation. Exosomes are small vesicles (30–150 nm in size) secreted by cells that play an essential role in intercellular communication (Zaborowski et al., 2015; Jiang et al., 2021), mediated by a class of proteins and nucleic acids carried within the exosomes that are transported from cell to cell (Zamani et al., 2019). Under disease conditions, fibroblasts, cardiomyocytes, macrophages, lymphocytes, and vascular endothelial cells communicate with each other through exosomes and their cargoes, ultimately leading to myocardial fibrosis (Jiang et al., 2021). In recent years, although exosomes have been reported to be involved in the diagnosis and treatment of a variety of diseases, their role in myocardial fibrosis is less well understood. Therefore, this review summarizes recent research on exosomes in relation to cardiac fibrosis, discussing the possible mechanisms through which exosomes influence the condition and the potential applications of this knowledge in the management of cardiovascular disease.

## Structure and Function of Exosomes

**FIGURE 1:**
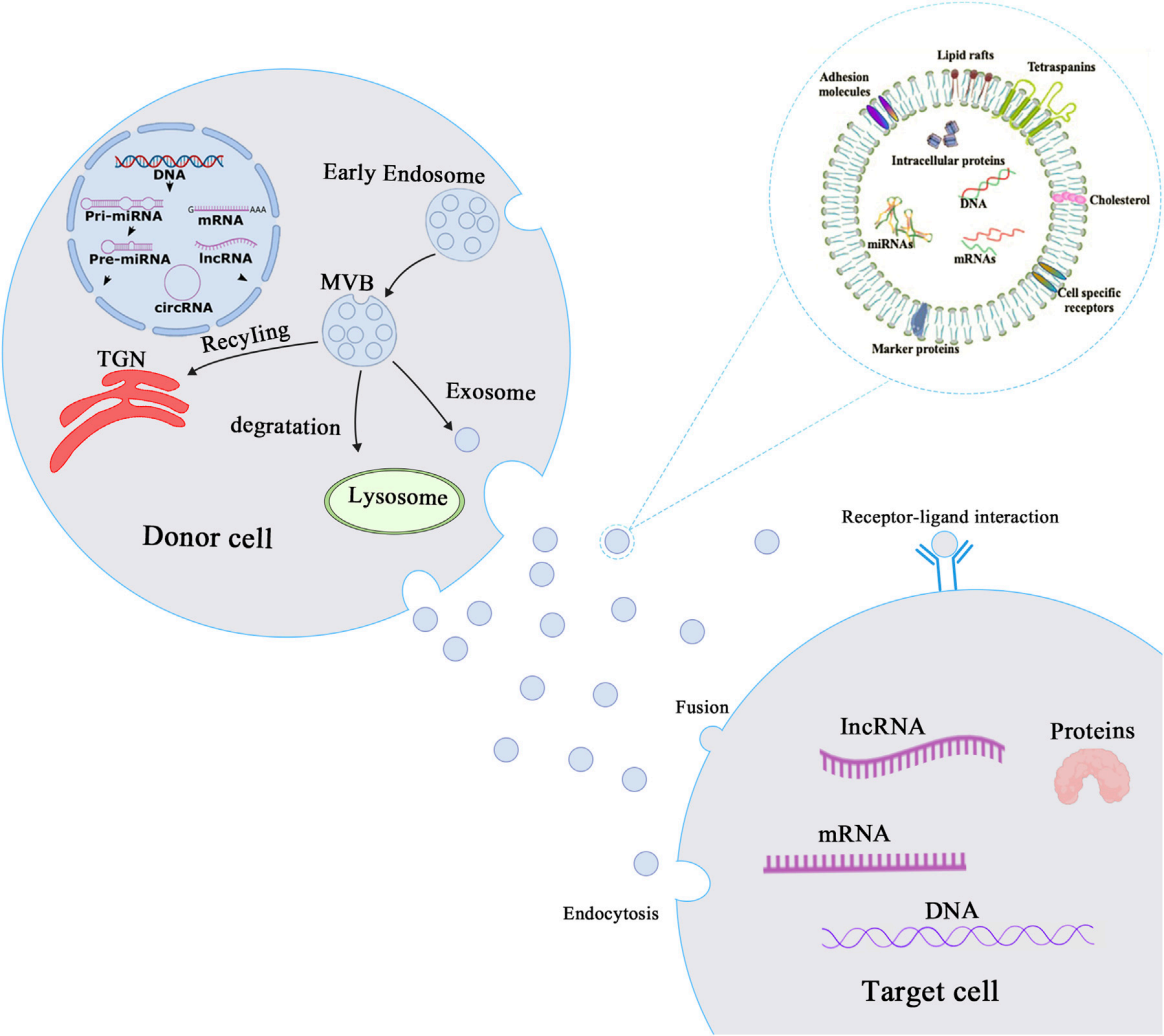
Biogenesis and mechanisms of action of exosomes. Biogenesis and mechanism of action of exosomes. The process of exosome production starts with the invagination of the cell membrane to form early endosomes, which bud inward to form intracellular multivesicular bodies (MVBs), and finally the outer membrane of MVB fuses with the cell membrane to release exosomes into the extracellular matrix. Exosomes then play a role in mediating intercellular communication through endocytosis, fusion or receptor-ligand interaction mechanisms.

Exosomes have a circular single-membrane structure and range between 30 and 150 nm in size. They are formed by a series of regulated processes such as endocytosis-fusion-exocytosis (Raposo & Stoorvogel, 2013; Zaborowski et al., 2015; Nguyen et al., 2016; Jiang et al., 2021). Early endosomes are formed by invagination of the cell membrane and subsequently mature into multivesicular bodies (MVBs) (Raposo & Stoorvogel, 2013; Yáñez-Mó et al., 2015). Exosomes are formed by MVB fusion with the cytoplasmic membrane and exosomal into the extracellular space, with the remaining MVBs recycled by the trans-Golgi network (TGN)or sent to lysosomes for degradation (Simons & Raposo, 2009; Théry et al., 2009; Bebelman et al., 2018; Doyle & Wang, 2019) (Figure 1). Exosomes were initially described in sheep reticulocytes in 1983 (Bobrie et al., 2011; Zhang & Yu, 2019) with the term “exosome” coined by Johnstone in 1987 (Viaud et al., 2008; Zhang & Yu, 2019). All mammalian cells have been found to secrete exosomes, and exosomes are commonly found in various body fluids, comprising cerebrospinal fluid, blood, and saliva (Pisitkun et al., 2004; Caby et al., 2005; Akers et al., 2013; Hornick et al., 2015; Backes et al., 2016; Kim et al., 2020). Although initially exosomes were believed to transport waste material from cells (Hessvik & Llorente, 2018), they have subsequently been shown to play important roles in a variety of biological and pathological processes. Exosomes are not only involved in intercellular material transport and information transfer, regulating cellular physiological activities but are also involved in all aspects of the body’s immune response, antigen presentation, cell migration, cell differentiation, and tumor invasion (Kalluri, 2016; Lindenbergh & Stoorvogel, 2018; Mashouri et al., 2019; Zhang & Yu, 2019; Daassi et al., 2020; Shen et al., 2020; Sung et al., 2021). Exosomes contain a wide variety of cargoes that are responsible for mediating intercellular communication and performing biological functions.

After uptake by target cells, mRNAs from the exosomal cargo can be translated into proteins, and mRNA expression can be regulated by microRNAs (miRNAs) at the post-transcriptional level (Figure 1) (Thum & Condorelli, 2015; Zhang et al., 2015; Piccoli et al., 2016; Yang et al., 2020). In addition, both exosomal cargoes and membranes contain numerous biologically active proteins, such as Rab proteins, annexins (regulating exosomal membrane exchange and fusion) (Monastyrskaya, 2018), heat shock proteins (HSP60, HSP70, HSP90), tetraspanin transmembrane proteins (CD9, CD63, and CD81), metabolic enzymes (such as GAPDH, enolase, and antioxidant enzymes), and adhesion factors (MFGE8, integrins) (Klinkert & Echard, 2016; Gao et al., 2019). With the advancement of research on exosomes and the application of advanced experimental techniques, exosomes are expected to become early diagnostic markers for several diseases and to be used as carriers of genes and drugs.

## Pathogenesis of Cardiac Fibrosis

**FIGURE 2:**
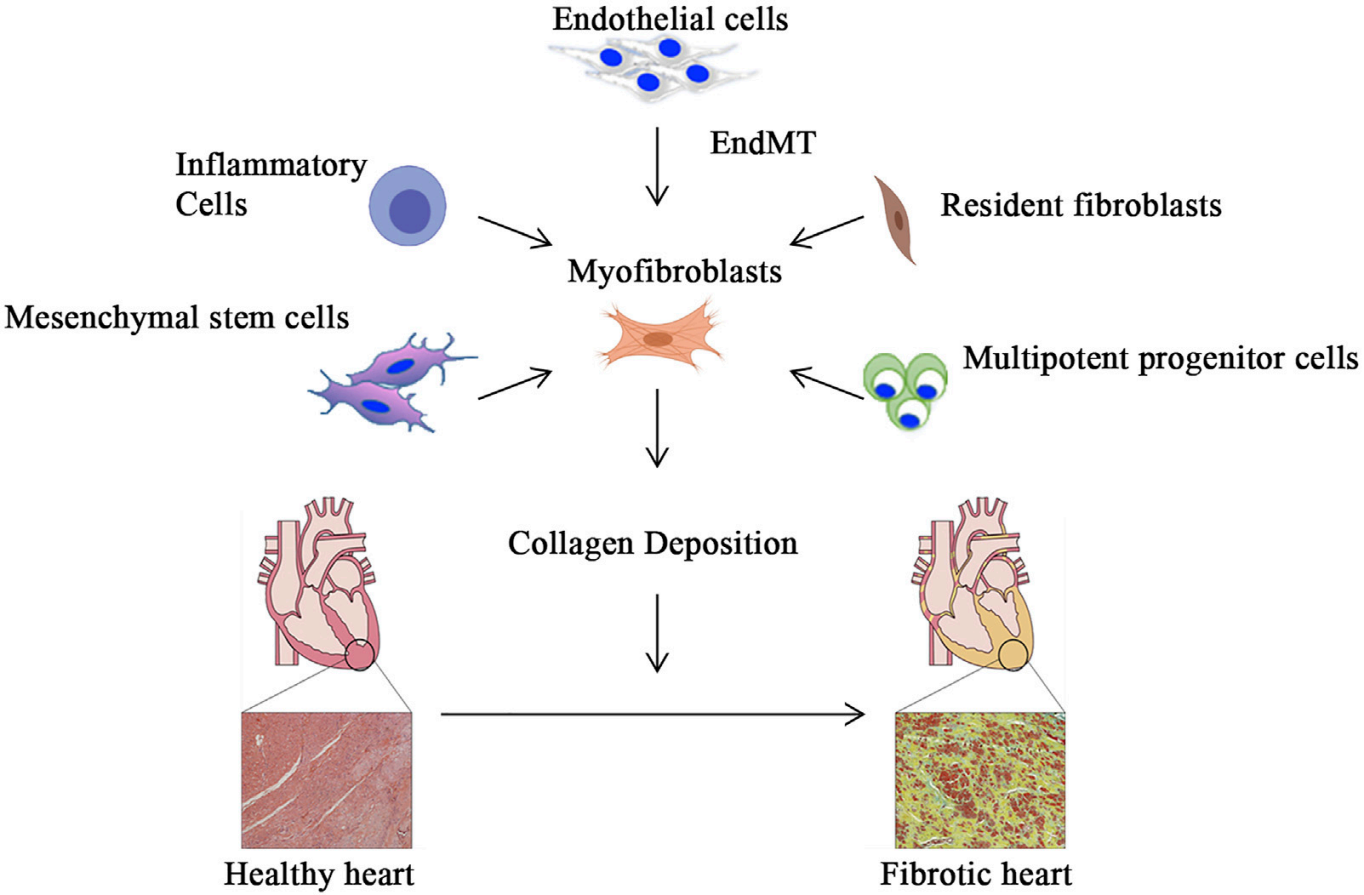
Myofibroblast activation and myocardial fibrosis. In addition to differentiation from resident fibroblasts, myofibroblasts can be derived from inflammatory cells, bone marrow-derived mesenchymal stem cells and pluripotent progenitor cells, and endothelial cells that undergo endothelial-mesenchymal transition. Subsequently, activated myofibroblasts secrete extracellular matrix, which gradually leads to the development of myocardial fibrosis.

Cardiac fibrosis is well-defined through remodeling of the myocardial interstitium, with enhanced numbers of myofibroblasts and collagen accumulation. This leads to abnormalities in cardiac function and metabolism and is commonly seen at the end stages of several cardiovascular diseases (Pathak et al., 2005; Frangogiannis, 2019, 2021). Cardiac fibrosis results in myocardial stiffness and reduced compliance of the ventricular wall, which severely affects the systolic and diastolic functions of the heart (Porter & Turner, 2009; Park et al., 2019). Cardiac fibrosis is usually attributed to one of two types: acute (or reparative) and chronic (or reactive) fibrosis. Since the adult mammalian heart has limited regenerating ability, the death of a significant proportion of cardiomyocytes causes reparative fibrosis. Dead myocardium activates the reparative process and is replaced by collagen-based scar after an acute myocardial infarction, which is necessary to safeguard the structural integrity of the ventricles and prevent cardiac rupture (Talman & Ruskoaho, 2016). In the absence of infarction, pathophysiologic factors such as pressure overload, volume overload, metabolic dysfunction, and aging can facilitate interstitial and perivascular fibrosis, and these changes are known as reactive fibrosis (Frangogiannis, 2019). Left ventricular pressure overload typically occurs as a result of systemic hypertension or aortic valve dysfunction, resulting in progressive interstitial and perivascular reactive fibrosis of the myocardium with a substantial proportion of noncollagenous matrix. Aging and abnormalities of glycolipid metabolism can also cause an interstitial myocardial fibrotic response, which is characterized by increased cardiac stiffness while the ejection fraction remains unaltered (Cavalera et al., 2014; Nattel, 2017; Bai et al., 2019). Cardiac fibroblasts are the major matrix-producing cells and represent one of the largest cell populations in normal mammalian hearts. They are closely involved in the maintenance of normal cardiac function as well as cardiac remodeling in pathological states (Camelliti et al., 2005; Tallquist, 2020). Fibroblasts in the fibrotic heart are a highly heterogeneous population of cells, and this heterogeneity is caused by their different origins. Resident fibroblasts are by far the most important source of myofibroblasts. In addition, myofibroblasts are also differentiated from endothelial cells, bone marrow-derived mesenchymal stem cells, and inflammatory cells (Figure 2) (Li et al., 2018). Although the conversion of cardiac fibroblasts into myofibroblasts is the most important initial step in all myocardial fibrosis, different etiologies trigger different molecular patterns of fibroblast activation that affect the pathological outcome of cardiac fibrosis.

The pathogenesis of myocardial fibrosis is complex and remains unclear. Fibrogenic signaling cascades are triggered by fibrogenic growth factors (including PDGFs and TGF-β), cytokines (such as TNFα, IL-1, IL-6, and IL-10), and neurohumoral pathway components that bind to cell surface receptors, activating downstream signaling cascades (Kong et al., 2014). Current research has revealed a close connection between the well-studied TGF-β/Smad signaling pathway and cardiac fibrosis that affects both the secretion and degradation of ECM components and myofibroblast differentiation (Khalil et al., 2017). The involvement of the innate immune system in the regulation of heart functioning and remodeling is well-documented (Baci et al., 2020). NLRP3 inflammasome activation and the levels of pro-inflammatory cytokine, comprising those of TNF-α, IL-1, and IL-6, have been found to be significantly elevated in several pathological cardiac conditions linked with fibrosis (Bracey et al., 2015; Siamwala et al., 2020). Louwe et al. demonstrated that transplantation of *Nlrp3* ^*−/−*^ bone marrow in mice after myocardial infarction attenuated cardiac remodeling compared with wild-type bone marrow, suggesting that the absence of the NLRP3 inflammasome in hematopoietic cells reduces adverse remodeling (Louwe et al., 2020). Members of the endothelin family of peptides are important regulators of cardiovascular function as well as the maintenance of basal vascular tone and cardiovascular system homeostasis (Barton & Yanagisawa, 2019; Miyauchi & Sakai, 2019). Recent studies have shown that ET-1 promotes fibroblast activation and collagen production and is an effective mediator of pro-fibrosis (Phosri et al., 2017; Zhang et al., 2021).

## Exosomes in Cardiac Fibrosis

There is mounting evidence of the involvement of exosomal intercellular communication in cardiac fibrosis (Qin et al., 2020; Jiang et al., 2021). Exosomes generated by different cells under the same disease condition or by the same cells under different disease conditions have positive or negative effects on fibroblast activation and cardiac fibrosis, a reflection of exosomal heterogeneity (Dutka et al., 2019; Hohn et al., 2021). Exosomal cargoes include proteins, lipids, mRNAs, miRNAs, and lncRNAs that are involved in paracrine signaling, underlying their significance in the regulation of cardiac fibrosis (Ranjan et al., 2019; Wei et al., 2021).

MiRNAs are a highly conserved subset of non-coding RNAs with lengths between 18 and 22 nucleotides. miRNAs interact with the 3’ UTR regions of their target mRNAs to modulate the expression of the latter (Bartel, 2004). (Yang et al., 2019)discovered upregulation of the expression of miR-208a in cardiomyocytes of rats after myocardial infarction; the miRNA was delivered to the fibroblasts by exosomes, leading to fibroblast proliferation and cardiac fibrosis (Yang et al., 2018). Furthermore, the application of an miR-208a antagomir was able to reverse this pro-fibrotic effect and ameliorate cardiac functions in post-myocardial-infarction rats. Previous investigations have demonstrated that the levels of miR-217 expression are enhanced in the hearts of cases suffering from chronic heart failure (Li et al., 2016). However, the role of miR-217 in myocardial fibrosis is unclear. Recently, Nie et al. (2018) found that cardiomyocyte-derived miR-217 was delivered by exosomes to act through a paracrine mechanism in fibroblasts, exacerbating pressure overload-induced myocardial fibrosis and cardiac dysfunction. In rat and mouse models of ischemic heart failure, miR-30d expression is elevated in cardiomyocytes and targets integrin α5 in fibroblasts *via* a paracrine mechanism, thus inhibiting fibroblast proliferation and fibrosis (Li et al., 2021). Song et al. found that direct injection of miR-21-rich exosomes into the myocardial infarct area of mice reduced scar formation in the infarct area and significantly improved cardiac function at 4 weeks (Song et al., 2019). Qiao et al. (2019) found that the impaired ability of exosomes derived from cardiac stromal cells of heart failure patients to promote endothelial tube formation and cardiomyocyte proliferation was attributed to dysregulated miR-21-5p expression. In contrast, stem cell-derived exosomes enriched in miR-21-5p improved cardiomyocyte function and attenuated myocardial fibrosis (Qiao et al., 2019). Moreover, Kang et al. found that miR-21 within human peripheral blood derived-exosomes increased cardiac fibrosis (Kang et al., 2019). Furthermore, miR-155, which is abundant in macrophage-derived exosomes, can aggravate cardiac fibrosis and inflammation by promoting the abnormal proliferation and differentiation of fibroblasts (Wang et al., 2017). The presence of increased numbers of CD4^+^ T cells in the heart is correlated with pathological cardiac remodeling and dysfunction (Savvatis et al., 2014). Cai et al. (2020) observed that exosomes originating from activated CD4+T cells accelerated post-ischemic cardiac fibrosis through the activation of myofibroblasts by exosome-derived miR-142-3p-WNT signaling. Recent literature suggests that exosomes are also able to modulate fibroblast function and reverse the pathological changes of myocardial fibrosis (Zhang et al., 2016; Yang et al., 2017). Exosomes produced by miR-126-overexpressing adipose-derived stem cells (ADSCs) were found to alleviate acute myocardial ischemic injury through the improvement of cardiac inflammation and fibrosis, suggesting that this could be a possible target for treating cardiac fibrosis (Luo et al., 2017). In recent years, several miRs (Table 1) that modulate fibroblast proliferation, differentiation, and cardiac fibrosis have been identified, and targeting these miRs in the exosome may help to diminish fibrosis and ameliorate cardiac functions.

**TABLE 1:** Exosomal miRNAs associated.

Name	Disease model	Target gene/pathway	Effects	References
miR-1246, miR-1290	Coronary artery ligation	ELF5, SP1	Angiogenesis↑, fibrosis↓	Huang et al. (2021)
miR-29c	Duchenne muscular dystrophy	TGFβ	Inflammation and fibrosis↓	Bier et al. (2018)
miR-21-5p	Coronary artery ligation	TIMP3	Ventricular remodeling↑	Dong et al. (2021)
miR-155	Uremic cardiomyopathy	FoxO3a	Fibrosis↑	Wang et al. (2020)
miR-27a, miR-28-3p, miR-34a	HF	Nrf2	Oxidative stress↑, Cardiac remodeling↑	Tian et al. (2018)
miR-19a-3p	Coronary artery ligation	Thrombospondin 1	Fibrosis↓	Gollmann-Tepeköylü et al. (2020)
miR-133a	Coronary artery ligation	Bim, Bmf, bFgf and Vegf	Cardiac fibrosis and hypertrophy↓	Izarra et al. (2014)
miR-10b-5p	MI	Smurf1,HDAC4	Cardiac fibroblast activatio↓	Liu et al. (2018)
miR-22	Coronary artery ligation	Mecp2	Fibrosis↓	Feng et al. (2014)
miR-24	Coronary artery ligation	MiR-24/Bim pathway	Fibrosis↓	Shao et al. (2020b)
miR-21-5p	Coronary artery ligation	Cdip1	Angiogenesis↑, fibrosis↓	Liao et al. (2021)
miR-30e	ISO-induced cardiac fibrosis	snai1/TGF-β pathway	Fibrosis↓	Zhang et al. (2017)
miR-146a-5p	Doxorubicin/trastuzumab-induced cardiac toxicity	Traf6, Smad4, Irak1, Nox4, and Mpo	Inflammation and fibrosis↓	Milano et al. (2020)
miR-150-5p	AngⅡ-induced cardiac fibrosis	EGR1	Fibrosis↓	Shen et al. (2019)
miR-218-5p, miR-363-3p	Coronary artery ligation	p53 and JMY	Fibrosis↓	Ke et al. (2021)
miR-290-295 cluster	Coronary artery ligation	Unknown	Cardiomyocyte survival and neovascularization↑, fibrosis↓	Khan et al. (2015)
miR-320a	HF	PIK3CA/Akt/mTOR signaling pathway	Myofibroblast proliferation↑, fibrosis↑	Wang et al. (2021b)
miR-26a	Uremic cardiomyopathy	Fox01	Collagen deposition↓, cardiac fibrosis↓	Wang et al. (2019)
miR-29b, miR-455	Diabetic cardiomyopathy	MMP-9	Fibrosis↓	Chaturvedi et al. (2015)
miR-378	Transverse aortic constriction (TAC)	MKK6/P38 MAPK pathway	Fibrosis↓	Yuan et al. (2018)
miR-19a	Coronary artery ligation	PTEN/Akt pathway	Fibrosis↓	Yu et al. (2015)
miR-210	Coronary artery ligation	MiR-210/HIF-1α	Fibrosis↓, Angiogenesis↑, Apoptosis↓	Zhu et al. (2018)
miR-92a	Coronary artery ligation	BMP2	Cardiac function and mouse survival↑, fibrosis↓	Ibrahim et al. (2019)
miR-133a	Coronary artery ligation	Bim, Bmf, bFgf, Vegf	Fibrosis↓, Hypertrophy↓, Angiogenesis↑	(Izarra et al., 2014; Jiang et al., 2021)
miR-146a-5p	Coronary artery ligation	EGR1/TLR4/NFk	Inflammation and fibrosis↓	Pan et al. (2019)
miR-425, miR-744	HF	TGF-β1	Fibrosis↓	Wang et al. (2018a)

Exosomal proteins have been shown to play a role in myocardial fibrosis regulation. Heat shock protein 90 (HSP90) in myocyte-derived exosomes was found to trigger STAT-3 signaling in cardiac fibroblasts, leading to excessive collagen synthesis and cardiac dysfunction during cardiac hypertrophy (Datta et al., 2017). Diabetic cardiomyocytes are more likely than their normal counterparts to release potentially damaging exosomes containing low amounts of HSP20. A study by Wang et al. (2016) reported that raised levels of HSP20 in exosomes in an HSP20-overexpressing transgenic mouse model significantly alleviated cardiac dysfunction and fibrosis resulting from streptozotocin treatment. Exosome-derived HSP70 levels are negatively correlated with aging, and downregulation of exosomal surface HSP70 levels may contribute to cardiac fibrosis (Yang et al., 2018). The signaling pathway of WNT performs a significant task in the development of cardiac fibrosis. Exosomes comprising WNT proteins are able to activate fibrosis-promoting WNT pathways in cardiac fibroblasts, suggesting a new strategy for the dissemination of fibrosis-promoting signals throughout the heart (Działo et al., 2019).

## Exosomes in the Diagnosis of Cardiac Fibrosis

Cardiac fibrosis results in structural and compliance alterations in the heart, ultimately leading to heart dysfunction and death (Kong et al., 2014; Sohns & Marrouche, 2020). Early identification and diagnosis of cardiac fibrosis are necessary to improve disease management and the prognosis and survival of patients. The diagnosis and classification of cardiac fibrosis rely largely on invasive tests, such as tissue biopsies, which are difficult to perform and put patients at risk, making the diagnosis of cardiac fibrosis very difficult. Fortunately, recent investigations have illustrated the significance of exosomal cargoes, especially miRNAs, for cardiac fibrosis and these can be used to diagnose myocardial fibrosis (Olson, 2014; Saadatpour et al., 2016; Poller et al., 2018). Exosomes represent novel biomarkers carrying disease-specific cargoes, are simple to obtain, and are stable in body fluids (Wang C. et al, 2021). Exosome-derived miRNAs should theoretically be better biomarkers than circulating miRNAs in plasma/serum as may be isolated from specific cell types, ensuring sensitivity and specificity (Jansen et al., 2014; Raju et al., 2020). A study by Wang et al. (2018a) reported that circulating exosomal miRNA-425 and miRNA-744 function as novel biomarkers that can predict cardiac fibrosis progression and heart dysfunction in patients with heart failure. Liu et al. (2019) found that miR-92a-3p within endothelial cell-derived exosomes was linked with atherogenesis (Liu et al., 2019) while Li et al. (2021) demonstrated that reduced expression of miR-30d in heart-derived exosomes was strongly related to deleterious cardiac remodeling and the expression of fibrosis- and inflammation-related genes in both rodent models and human subjects (Li et al., 2021). Jansen found that miR-126 and miR-199a in exosomes were found to be associated with cardiovascular events in stable coronary artery heart disease, rather than free miR-126 and miR-199a in plasma (Jansen et al., 2014). The increased levels of miR-1, and miR-133a were associated with acute MI, unstable angina pectoris, or Takotsubo cardiomyopathy (Barile et al., 2017). However, extensive studies in large groups of human patients are required before any final conclusions can be reached.

Additionally, exosomal proteins may also be associated with cardiac fibrosis. Overexpression of HIF-1α in exosomes from mesenchymal stem cells reduced cardiac fibrosis through the promotion of neovessel formation in rats after myocardial infarction (Sun et al., 2020). (Yang et al., 2019) found that HSP70 on the surface of serum exosomes was negatively associated with aging and was implicated in cardiac fibrosis, suggesting that it may be a promising diagnostic target for aging-related myocardial fibrosis (Yang et al., 2018). Therefore, exosomes are potentially effective diagnostic markers for the diagnosis and etiological classification of the early stages of cardiac fibrosis.

## Exosomes as Targets for the Treatment of Cardiac Fibrosis

Exosomes have been recently investigated as potential biotherapeutics and drug delivery vectors for the treatment of cardiac fibrosis. Many reports have suggested that exosomes can function as therapeutic agents and drug delivery systems (El-Andaloussi et al., 2012; Arrighetti et al., 2019; Shao J. et al, 2020; Liang et al., 2021). As exosomes carry specific cargoes of functional nucleic acids and proteins derived from particular cell types, it is likely that they may be used as therapeutic drugs. Milano et al. found that miR-146a-5 is significantly enriched in exosomes derived from resident cardiac mesenchymal progenitor cells, and intravenous administration of these exosomes could attenuate doxorubicin/trastuzumab-induced cardiotoxicity and alleviate cardiac fibrosis (Milano et al., 2020). Exosomes from cardiosphere-derived cells enriched with miR-92a attenuated myocardial fibrosis and improved survival in mice with myocardial infarction (Ibrahim et al., 2019). Previous studies have identified that miR-26a regulates extracellular matrix production and overexpression of miR-26a can inhibits cardiac fibrosis caused by diseases such as myocardial infarction and chronic kidney disease (CKD) (Wei et al., 2013; Wang et al., 2019; Chiang et al., 2020). Wang et al. (2019) engineered an exosome vector containing Lamp2b (an exosomal membrane protein gene fused with a muscle-specific surface peptide that targets muscle delivery) and transfected it into muscle satellite cells overexpressing miR-26a to generate miR-26a-enriched exosomes, which were subsequently injected into mouse tibialis anterior muscle. The results showed that miR-26a inhibited myocardial fibrotic lesions by reducing ECM production through inhibition of Fox01, which provides a possible therapeutic strategy for the use of exosome delivery of miR-26a for the treatment of CKD complications. In addition, Yao et al. (2021) advanced a minimally invasive exosome spray (EXOS) on the basis of the mesenchymal stem cell exosomes and biomaterials that can be used to reduce cardiac fibrosis in a mouse model of acute myocardial infarction.

Furthermore, exosomes can also act as drug carriers, thus enabling more efficient delivery of drugs to target cells. The natural compound curcumin has both anti-inflammatory and anti-fibrotic properties (He et al., 2015; Yu et al., 2019; Zhao et al., 2021). Sun et al. used exosomes for the targeted delivery of curcumin to inflammatory cells, significantly reducing inflammatory factor levels (Sun et al., 2010). In addition, they also demonstrated that curcumin delivered by exosomes had higher concentrations in the blood. With the improvement of technological tools, molecular cloning and lentiviral packaging techniques have been applied to the treatment of myocardial fibrosis. Wang et al. (2018b) designed engineered exosomes containing an ischemic myocardium-targeting peptide, which significantly reduced inflammation and fibrosis in the ischemic heart region in a mouse model of myocardial infarction. Plant-derived exosome-like nanoparticles (ELNs) have been shown to also prevent and treat a variety of diseases, with functions closely related to the plant of origin (Teng et al., 2018; Cong et al., 2022). ELNs have lipid membrane structures that can also be used as natural or engineered carriers for drug loading and delivery (Karamanidou & Tsouknidas, 2021). Moreover, ELNs are cost-effective and easy to obtain, and have great potential for development and application. In brief, the above studies provide robust evidence for the use of exosomes as antifibrotic therapy.

## Conclusion

Cardiac fibrosis is a hallmark of cardiac remodeling and a cause of clinical disease. The search for ways to slow, stop, or even reverse the progression of myocardial fibrosis has generated widespread interest. Cardiac fibrosis occurs frequently in the course of several cardiovascular diseases and can result in heart failure and death. Over the past few years, many extensive and in-depth studies of exosomes have provided important new insights that have led to a clearer understanding of both exosomal structures and actions. There is mounting evidence that exosomes play significant roles in cardiac fibrosis, and they can thus be useful as biomarkers and drug delivery vehicles for the diagnosis and treatment, respectively, of cardiac fibrosis. Although no exosome-based treatment for cardiac fibrosis has been clinically realized at this stage, the application of exosomes has already displayed a strong therapeutic potential, and thus further research is needed in the future. In addition, several unanswered questions remain to be addressed, such as 1) How to efficiently get samples with the required cargo? 2) What factors influence exosome biogenesis in cardiac fibrosis? 3) What are the long-term effects and possible concomitant adverse effects of exosome therapy for cardiac fibrosis?
